# Glucose Metabolic Brain Networks in Early-Onset vs. Late-Onset Alzheimer's Disease

**DOI:** 10.3389/fnagi.2016.00159

**Published:** 2016-06-30

**Authors:** Jinyong Chung, Kwangsun Yoo, Eunjoo Kim, Duk L. Na, Yong Jeong

**Affiliations:** ^1^Department of Bio and Brain Engineering, Korea Advanced Institute of Science and TechnologyDaejeon, South Korea; ^2^KAIST Institute for Health Science and Technology, Korea Advanced Institute of Science and TechnologyDaejeon, South Korea; ^3^Department of Neurology, School of Medicine and Medical Research Institute, Pusan National UniversityBusan, South Korea; ^4^Department of Neurology, Samsung Medical Center, Sungkyunkwan University School of MedicineSeoul, South Korea; ^5^Neuroscience Center, Samsung Medical CenterSeoul, South Korea

**Keywords:** FDG-PET, early-onset, Alzheimer disease, metabolic connectivity, network

## Abstract

**Objective:** Early-onset Alzheimer's disease (EAD) shows distinct features from late-onset Alzheimer's disease (LAD). To explore the characteristics of EAD, clinical, neuropsychological, and functional imaging studies have been conducted. However, differences between EAD and LAD are not clear, especially in terms of brain connectivity and networks. In this study, we investigated the differences in metabolic connectivity between EAD and LAD by adopting graph theory measures.

**Methods:** We analyzed ^18^F-fluorodeoxyglucose-positron emission tomography (FDG-PET) images to investigate the distinct features of metabolic connectivity between EAD and LAD. Using metabolic connectivity and graph theory analysis, metabolic network differences between LAD and EAD were explored.

**Results:** Results showed the decreased connectivity centered in the cingulate gyri and occipital regions in EAD, whereas decreased connectivity in the occipital and temporal regions as well as increased connectivity in the supplementary motor area were observed in LAD when compared with age-matched control groups. Global efficiency and clustering coefficients were decreased in EAD but not in LAD. EAD showed progressive network deterioration as a function of disease severity and clinical dementia rating (CDR) scores, mainly in terms of connectivity between the cingulate gyri and occipital regions. Global efficiency and clustering coefficients were also decreased along with disease severity.

**Conclusion:** These results indicate that EAD and LAD have distinguished features in terms of metabolic connectivity, with EAD demonstrating more extensive and progressive deterioration.

## Introduction

Alzheimer's disease (AD) is the most common form of dementia. Every year, the number of AD cases increases exponentially, paralleling the worldwide increase in the elderly population. Many studies have investigated the pathogenesis, diagnosis, treatment, and prevention of AD; however, there are many issues yet to be elucidated. Recently, resting-state functional magnetic resonance imaging (fMRI) studies have been employed for this purpose (Kochan et al., 2010). For example, resting functional connectivity in the default mode network (DMN) is altered in patients with AD compared to healthy adults (Amaducci et al., 1986; Greicius et al., 2003; Wu et al., 2011; Sohn et al., 2014). This suggests that functional connectivity changes within certain brain networks could be a useful biomarker for the early detection of AD.

AD can be divided into early- and late-onset AD (LAD), based on symptom onset age before or after the arbitrary cut-off age of 65 (Amaducci et al., 1986). LAD is regarded as “typical” AD, which is accompanied by significant memory impairment. Conversely, early-onset AD (EAD) presents with fewer memory problems but more significant visuospatial problems, aphasia, apraxia, and agnosia (Wallin and Blennow, 1992; Jacobs et al., 1994; Hodges et al., 2006; McMurtray et al., 2006; Shinagawa et al., 2007; Koedam et al., 2009; Migliaccio et al., 2009; Kim et al., 2010; Smits et al., 2012; Van Vliet et al., 2012). Several studies showed different topographic changes in brain structure and/or metabolism in EAD (Schreiter-Gasser et al., 1993; Ishii et al., 2005; Kim et al., 2005; Shiino et al., 2006; Frisoni et al., 2007; Karas et al., 2007; Shiino et al., 2008; Rabinovici et al., 2010; Canu et al., 2012). One study showed that the parietal areas are particularly vulnerable to metabolic impairment in EAD (Kim et al., 2005). Another study showed that cortical atrophy is most prominent in the parietal and occipital cortex among EAD patients while the medial temporal lobe is the most affected region in LAD (Schreiter-Gasser et al., 1993; Sakamoto et al., 2002; Ishii et al., 2005; Shiino et al., 2006; Frisoni et al., 2007; Karas et al., 2007; Shiino et al., 2008; Canu et al., 2012). These findings may indicate distinct pathophysiology between EAD and LAD.

Although several functional neuroimaging studies have examined topographic changes, few studies have investigated EAD by conducting an analysis of brain connectivity and networks. Two Studies have investigated functional brain organization in EAD using fMRI (Adriaanse et al., 2014; Gour et al., 2014). The study (Adriaanse et al., 2014) found that functional connectivity in the auditory, sensorimotor, dorsal visual, and DMN regions was diminished in EAD compared to LAD. The authors concluded that functional brain organization was more widely disrupted in EAD compared to LAD. This result supports findings from previous topological studies using fluorodeoxyglucose-positron emission tomography (FDG-PET) (Kim et al., 2005) and provides evidence for functional differences between EAD and LAD.

These clinical and topological differences indicate that LAD and EAD may be different entities and thus require different remedies. Therefore, the identification of distinct features in EAD is important and we propose that metabolic network properties can provide this information. To our knowledge, there has not yet been a study to differentiate the metabolic network features of EAD and LAD. Metabolic network analyses have an advantage over resting fMRI connectivity assessments since the former reflect neuronal activity while the latter reflect indirect hemodynamic changes.

The goal of the current study is to investigate the differences in metabolic connectivity and neural networks between EAD and LAD using FDG-PET. Specifically, we attempt (i) to determine which brain regions are affected in terms of glucose metabolic connectivity in EAD and LAD, (ii) to examine the changes based on dementia severity, and (iii) to assess the changes in network parameters between EAD and LAD according to dementia severity.

## Methods

### Subjects

The present study used the same data set as our previous study (Kim et al., 2005). FDG-PET images from 46 patients with LAD, 74 patients with EAD, 20 young, and 13 old age-matched controls were recruited. Patients were diagnosed with AD at the Memory Disorder Clinic at the Samsung Medical Center using the National Institute of Neurological and Communicative Disorders and Stroke and the Alzheimer's disease and Related Disorders Association (NINCDS-ADRDA) criteria. Controls' cognition was confirmed to be within normal limits as assessed by the Mini Mental State Exam (MMSE; mean 29.2 ± 0.8) and the Seoul Neuropsychological Screening Battery (SNSB) (Kang and Na, 2003). Patients with the familial form of AD were excluded. AD onset age was determined by a caregiver's report during the patient's first visit to the memory disorder clinic. Table 1A shows the demographic and clinical information for each group. Normal controls were divided into two age-matched groups: old controls (age 71.5 ± 2.0) and young controls (age 56.4 ± 4.9). The EAD group was also stratified by the CDR: 23 patients had a CDR = 0.5, 25 had a CDR = 1, 17 has a CDR = 2, and 9 had a CDR = 3. Demographics and MMSE scores for the EAD subgroups are shown in Table 1B. The results of neuropsychological tests in early onset vs. late onset Alzheimer's disease are accessible through a previous study (Kim et al., 2005)

**Table 1A:** **Demographics and clinical information of subjects**.

	**Early-onset group**			**Late-onset group**		
	**Alzheimer's disease (*n* = 74)**	**Control (*n* = 20)**	***p* value**	**Alzheimer's disease (*n* = 46)**	**Control (*n* = 13)**	***p*-value**
Age at onset	55.7 ± 5.4	−	−	69.6 ± 3.1	−	−
Age at examination	59.1 ± 5.7	56.0 ± 4.9	0.053	72.8 ± 3.6	71.5 ± 2.0	0.084
Sex (female %)	66.2%	55.0%	0.354	71.7%	30.8%	0.01
Duration of education (years)	10.6 ± 4.9	11.6 ± 4.2	0.395	9.3 ± 5.3	11.1 ± 3.9	0.283
CDR	1.3 ± 0.9	−	−	1.3 ± 0.8	−	−
MMSE scores	17.4 ± 7.1	29.3 ± 0.7	0.000	18.5 ± 7.1	29.0 ± 0.9	0.000

*MMSE, Mini-mental State Examination; CDR, Clinical Dementia Rating*.

**Table 1B:** **Demographics and clinical information of subgroups**.

**EARLY-ONSET AD**				
**Group (n)**	**CDR 0.5 (23)**	**CDR 1 (25)**	**CDR 2 (17)**	**CDR 3 (9)**
Age at onset	57.0 ± 6.0	56.0 ± 4.8	53.0 ± 5.6	56.0 ± 4.4
Age at examination	59.6 ± 6.3	59.5 ± 5.0	56.9 ± 6.0	60.9 ± 5.3
Sex (female %)	70.0	64.0	70.6	55.6
Duration of education (years)	10.8 ± 4.5	11.7 ± 5.4	8.7 ± 4.7	10.3 ± 4.7
MMSE scores	23.0 ± 3.9	19.0 ± 4.0	14.1 ± 4.4	4.4 ± 3.6
**LATE-ONSET AD**				
**Group (n)**	**CDR 0.5 (13)**	**CDR 1 (16)**	**CDR 2 (13)**	**CDR 3 (4)**
Age at onset	70.0 ± 3.6	69.0 ± 3.2	70.0 ± 2.5	69.0 ± 6.2
Age at examination	72.0 ± 3.8	72.3 ± 3.7	73.5 ± 3.04	74.5 ± 6.9
Sex (female %)	61.5	81.3	84.6	25.0
Duration of education (years)	9.2 ± 5.7	9.3 ± 5.4	8.1 ± 5.7	10.6 ± 5.3
MMSE scores	23.8 ± 3.2	20.7 ± 4.1	13.3 ± 6.4	8.8 ± 8.7

*MMSE, Mini-mental State Examination; CDR, Clinical Dementia Rating*.

### FDG-PET data acquisition and processing

FDG-PET images were acquired for 30 min after an intravenous injection of 4.8 MBq/kg FDG using a General Electric Advance PET scanner. Participants stayed in a dimly lit room with their eyes closed. The in-plane and axial resolution was 4.9 × 3.9 mm full-width at half maximum (FWHM).

PET images were analyzed using SPM8 (Wellcome Department of Cognitive Neurology, Institute of Neurology, London, UK) (Friston et al., 1995) in MATLAB 7.10.0 (R2010a) (MathWorks Inc., Sherborn, MA). PET data were initially preprocessed. First, all PET images were spatially normalized into the Montreal Neurological Institute (MNI) template (MNI, McGill University, Montreal, Canada) to minimize inter-subject structural variability. Second, smoothing was performed by convolution using an isotropic Gaussian kernel with a 16-mm FWHM. Third, PET images went through one more normalization step to adjust for FDG intensity. Each voxel was normalized by the mean intensity of the cerebellum, which is known to be the least affected region in AD. The cerebellar areas were chosen using the Automated Anatomical Labeling (AAL) template.

### Region-of-interest-based metabolic connectivity analysis

Subject-series FDG-PET data were obtained for each group. Similar to correlations in the fMRI time-series data, Pearson's correlations were likewise calculated. The areas were defined using the AAL template. These interregional metabolic correlations were regarded as the metabolic connectivity metric (Lee et al., 2008). We calculated the metabolic connectivity matrices based on 90 brain regions using the AAL template after excluding the cerebellum.

To test the statistical significance of connectivity differences between groups, we used a non-parametric permutation test. The difference in metabolic connectivity between the 90 regions in paired groups was tested. All subjects in each group were resampled and 10,000 permutations were analyzed. Metabolic connectivity was calculated and 10,000 subtracted metabolic connectivity matrices between groups were created. Finally, an actual subtracted metabolic connectivity matrix was tested and significant differences between groups were obtained.

### Network analysis

We used graph theory to create parameters for a network analysis. Two network parameters—the clustering coefficient and global efficiency—were obtained.

The clustering coefficient (C) is a global parameter that indicates the mean of local interconnectivity in the network. *C*_*i*_ is the local clustering coefficient of node *i*, defined as the number of connections between the node and neighboring nodes divided by all possible connections. Neighboring nodes have at least one connection with node *i*. The clustering coefficient (C) for the whole network (G) is defined as the average of all *C*_*i*_*s*:

$$\text{C}=\frac{1}{\text{N}}\;\sum_{i\;\in \;G}C_{i}$$

where N represents the number of nodes.

Global efficiency (E) is also a parameter that represents the efficiency of information flow in the network. The global efficiency (E) is expressed as:

$$\text{E}=\frac{1}{\text{N}(\text{N}-1)}\;\sum_{\begin{array}{c} i,\text{​}j\text{​ }\in \text{ ​}G\\ i\neq j \end{array}}\;\frac{1}{d_{ij}}$$

Where *d*_*ij*_ is defined as the number of connections along the shortest path connecting nodes *i* and *j*.

To analyze these network parameters, we assumed that the brain is a complex system and should have properties consistent with a small-world network (Bassett and Bullmore, 2006). Small-world topology is a well-fitted model for brain networks because this can support both segregated/specialized and distributed/integrated information processing which are distinct features of brain. Thus, small-worldness tests were performed to choose appropriate network densities which maintain the properties of small-worldness. The small-worldness parameter (σ), which indicates the degree of small-worldness, was acquired by a combination of (C) and the shortest path of length (L) compared to a random network. The small-world network has a small average shortest path length, nearly the same as the random network ($\lambda =\frac{{\text{L}}^{\text{real}}}{{\text{L}}^{\text{rand}}}\sim 1$), and has a higher cluster coefficient value than the random network ($\gamma =\frac{{\text{C}}^{\text{real}}}{{\text{C}}^{\text{rand}}}>>1$). Thus, the small-world parameter is defined as: $\sigma =\frac{\gamma }{\lambda }>1$. For each density, random networks were made by the edge randomization between nodes while graph density was maintained. The mean values of parameters were used from 1000 randomizations.

To analyze network parameters, we used the same 10,000 permutation sets from the metabolic connectivity analysis. We generated 10,000 permuted network sets for any two groups and calculated the difference of each parameter in each network set. This subtracted value was statistically tested by non-parametric permutation. All network parameters were calculated using a weighted connectivity matrix.

## Results

### Glucose hypometabolism and metabolic connectivity differences between EAD and LAD

**Figure 1:**
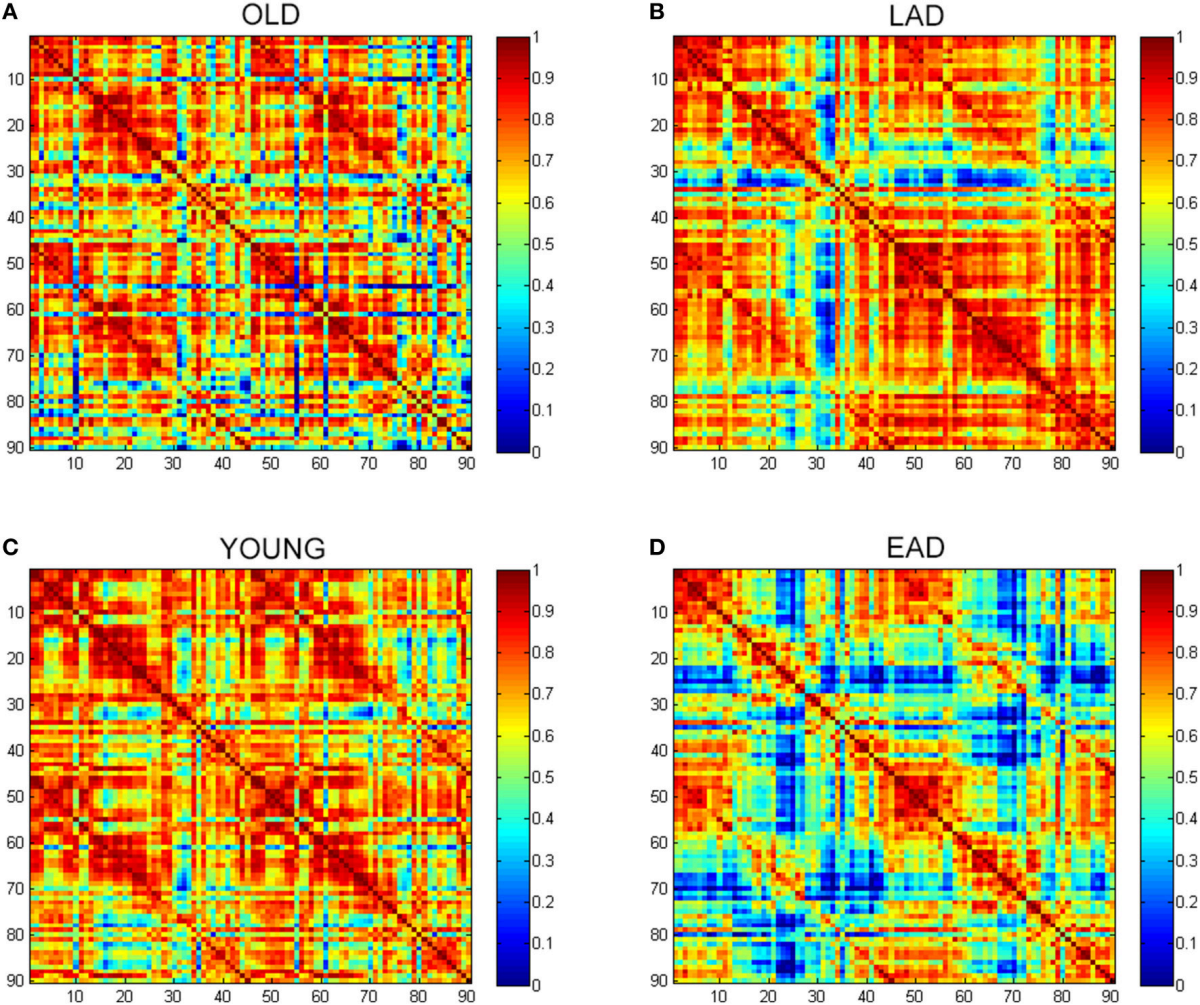
**Glucose metabolic connectivity matrix**. A glucose metabolic connectivity matrix was calculated for each group. Each axis represents 90 brain regions based on the Automated Anatomical Labeling (AAL) template. Glucose metabolic connectivity is determined via a Pearson correlation between two regions corresponding to a (x, y) coordinate. **(A)** Old control group (OLD), **(B)** Late-onset Alzheimer's disease (LAD), **(C)** Young control group (YOUNG), **(D)** Early-onset Alzheimer's disease (EAD).

**Figure 2:**
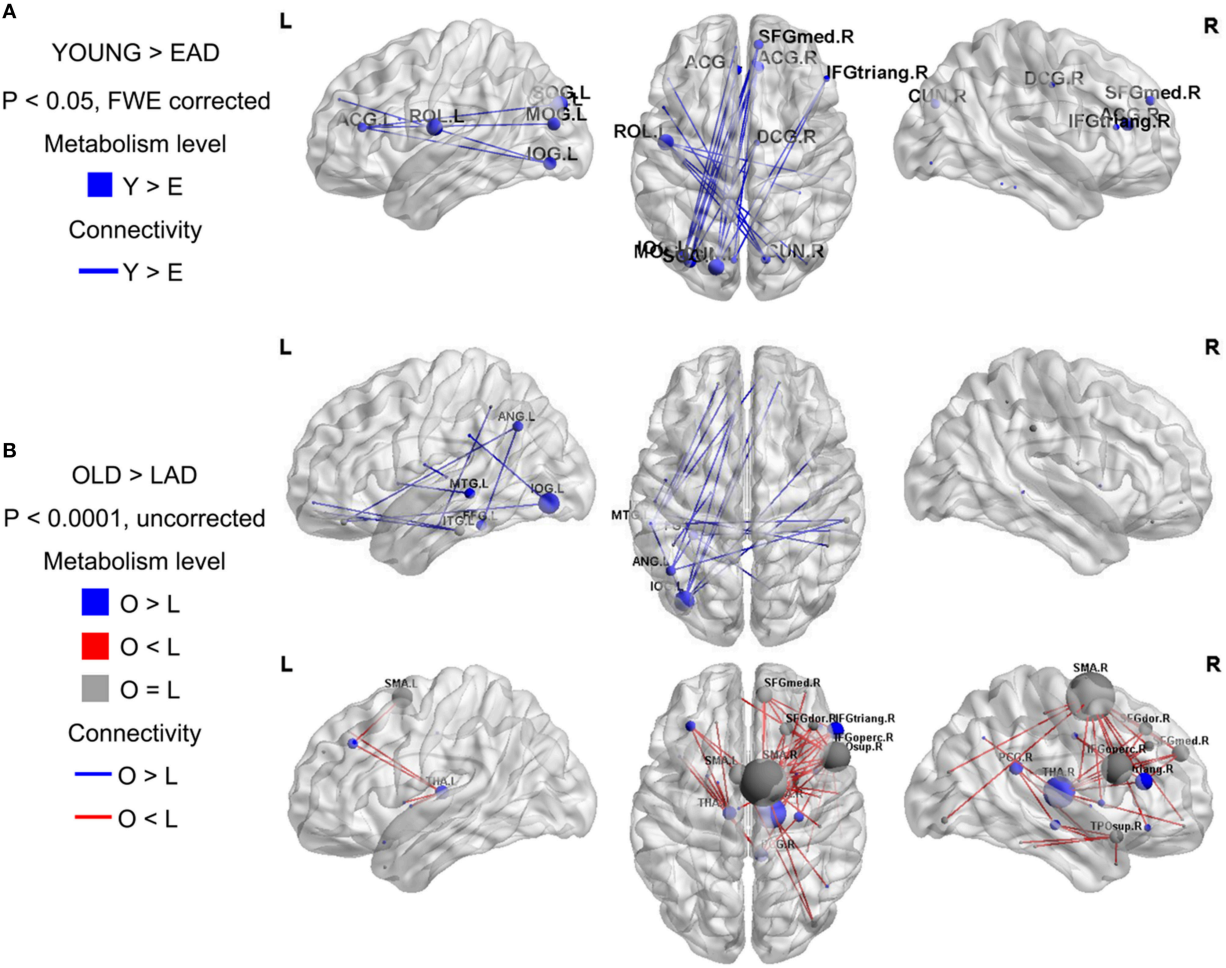
**Metabolic connectivity differences between the Alzheimer's disease (AD) and control groups**. The node color indicates differences in the metabolic levels while its size indicates the differences in metabolic connectivity. The line color indicates differences in metabolic connectivity. Nodes larger than three are presented in the figure as an acronym. The early-onset Alzheimer's disease (EAD) group has decreased metabolic connectivity compared to young control (YOUNG) group (*p* < 0.05, FWE corrected) **(A)**. The late-onset Alzheimer's disease (LAD) group has no differences in metabolic connectivity compared to the old control (OLD) group (*p* < 0.05, FWE corrected). However, LAD has both decreased and increased metabolic connectivity at a lower threshold (*p* < 0.0001, uncorrected) **(B)**.

**Figure 3:**
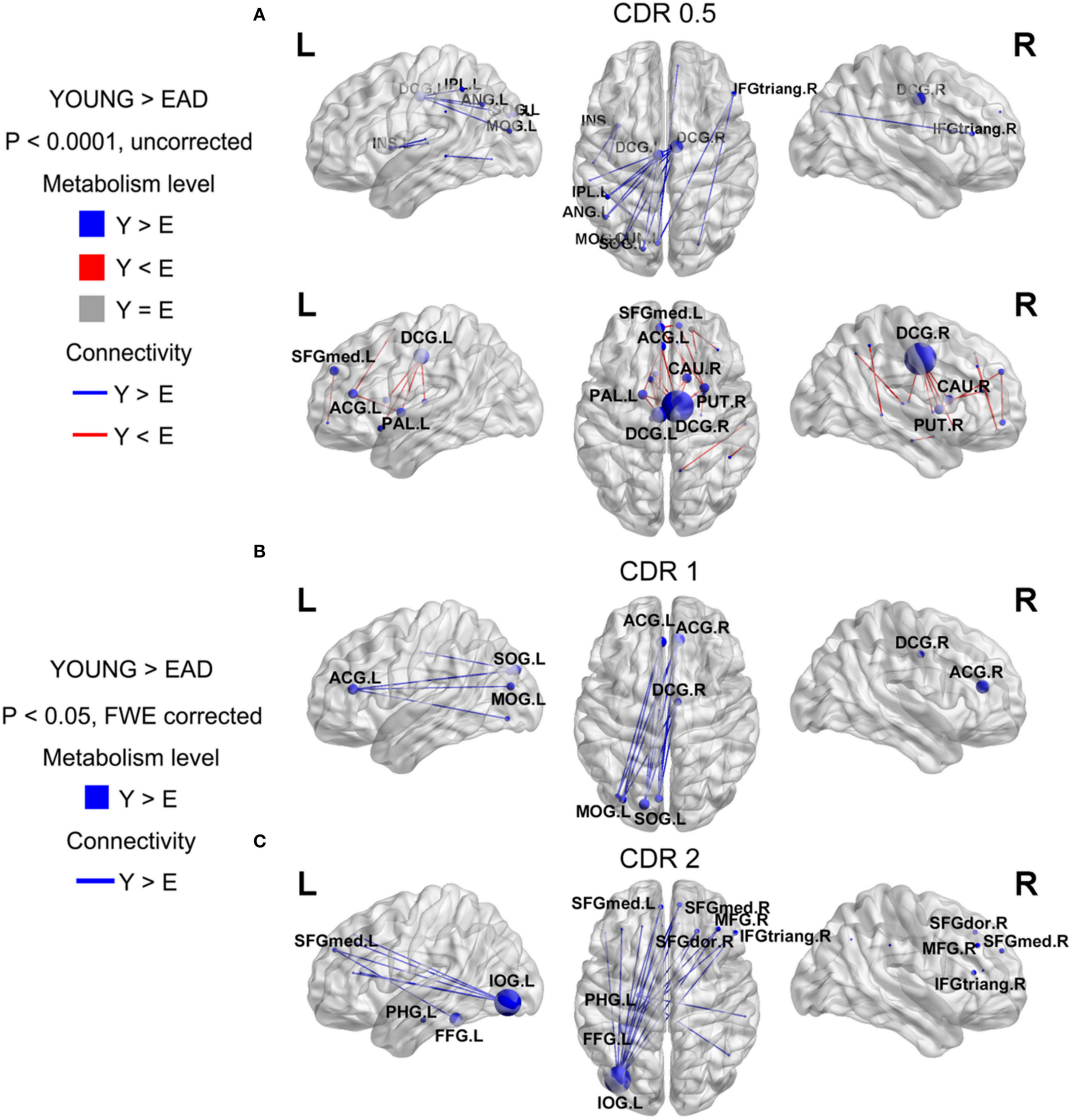
**Metabolic connectivity differences between the early-onset Alzheimer's disease (EAD) subgroups based on CDR scores**. The node color indicates differences in metabolic level. The node size indicates the differences in metabolic connectivity. The line color indicates differences in metabolic connectivity. Nodes larger than three are presented as an acronym. The CDR = 0.5 subgroup has no differences in metabolic connectivity compared to the old control (OLD) group (*p* < 0.05, FWE corrected) **(A)**. However, this group has both decreased and increased metabolic connectivity with a lower threshold (*p* < 0.0001, uncorrected). The other subgroups only show decreased metabolic connectivity compared to young controls (YOUNG) at an adjusted threshold (*p* < 0.05, FWE corrected) **(B,C)**.

To examine the overall connectivity pattern, metabolic connectivity matrices for each group were acquired by calculating metabolic correlation coefficients across each region (Figure 1). The EAD and LAD groups had significantly lower metabolic connectivity between certain regions compared to the control groups. To obtain topographic information, connectivity was rendered on a template (Figures 2, 3) (Xia et al., 2013). Metabolic connectivity between the left occipital area and cingulate gyri was significantly decreased in the EAD group (Figure 2A). However, the LAD group showed no significant differences in metabolic connectivity (*p* < 0.05, FWE corrected). Interestingly, some connectivity centered on the supplementary motor area was increased in the LAD group under a less conservative statistical threshold (*p* < 0.0001, uncorrected) (Figure 2B). This group also showed decreased connectivity between the left occipital area and the temporal and medial frontal areas (*p* < 0.0001, FWE corrected) (Figure 2B).

We compared metabolic connectivity differences in the LAD and EAD groups according to dementia severity based on the CDR. The LAD group did not show any significant differences in connectivity according to dementia severity. In the EAD group, the metabolic connectivity analysis revealed divergent but successive changes in connectivity with an increase in CDR (Figure 3). In the CDR = 0.5 subgroup, connectivity was centered on the dorsal cingulate gyrus with decreases in the left occipital and left parietal regions and with increases in the dorsal cingulate gyrus and subcortical regions (*p* < 0.0001, uncorrected) (Figure 3A). For the CDR = 1 subgroup, connectivity between the left occipital regions, anterior cingulate gyrus, and dorsal cingulate gyrus was decreased (*p* < 0.05, FWE corrected) (Figure 3B). In the CDR = 2 subgroup, connectivity was centered on the inferior occipital gyrus with decreases in the medial frontal regions and anterior cingulate gyrus (*p* < 0.05, FWE corrected) (Figure 3C).

### Glucose metabolic network property differences between EAD and LAD

**Figure 4:**
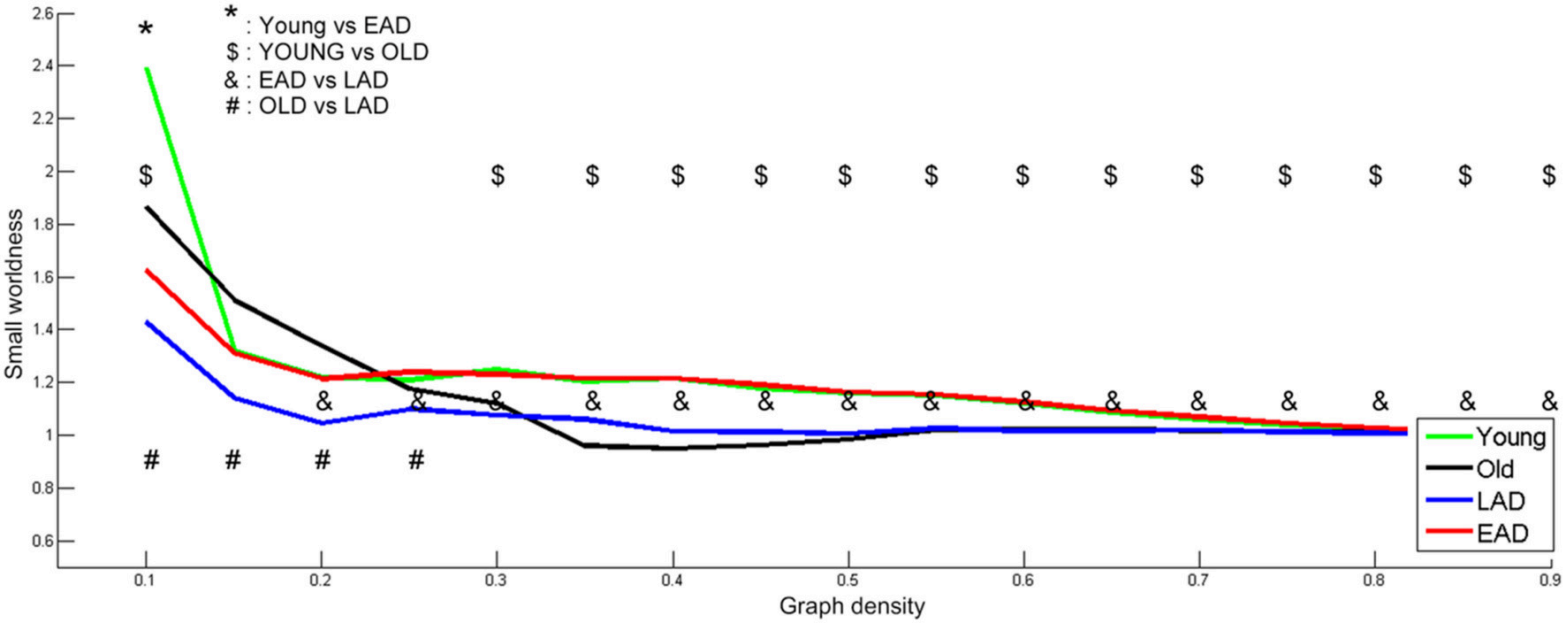
**Small-worldness parameters between the Alzheimer's disease (AD) and control groups**. A non-parametric test was used to find differences in network density. For small-worldness calculations, 10,000 random permutations were used. A significant network density period was determined at a threshold of *p* < 0.001 (uncorrected). Each comparison pair is a given different notational value. OLD, older control group; YOUNG, younger control group.

To determine optimal network density, the small-worldness characteristic was calculated based on whole densities (Figure 4). We examined network parameters within a density interval of 10–90% whose small-worldness value was over 1, except for the old control group. The density interval was divided into 17 parts with a 5% gap. Old controls had lower small-worldness (< 1) values within the 35-55% interval; thus, statistical tests were not performed for this range within the old control group. The young controls and the EAD groups had no differences in small-worldness values throughout most of the density ranges (15–90%). LAD and old control groups had lower small-worldness values than the other two groups throughout most of the density ranges (20–90%, 30–90%). However, the LAD group had lower small-worldness values than the old controls at the low-density ranges (10–25%).

**Figure 5:**
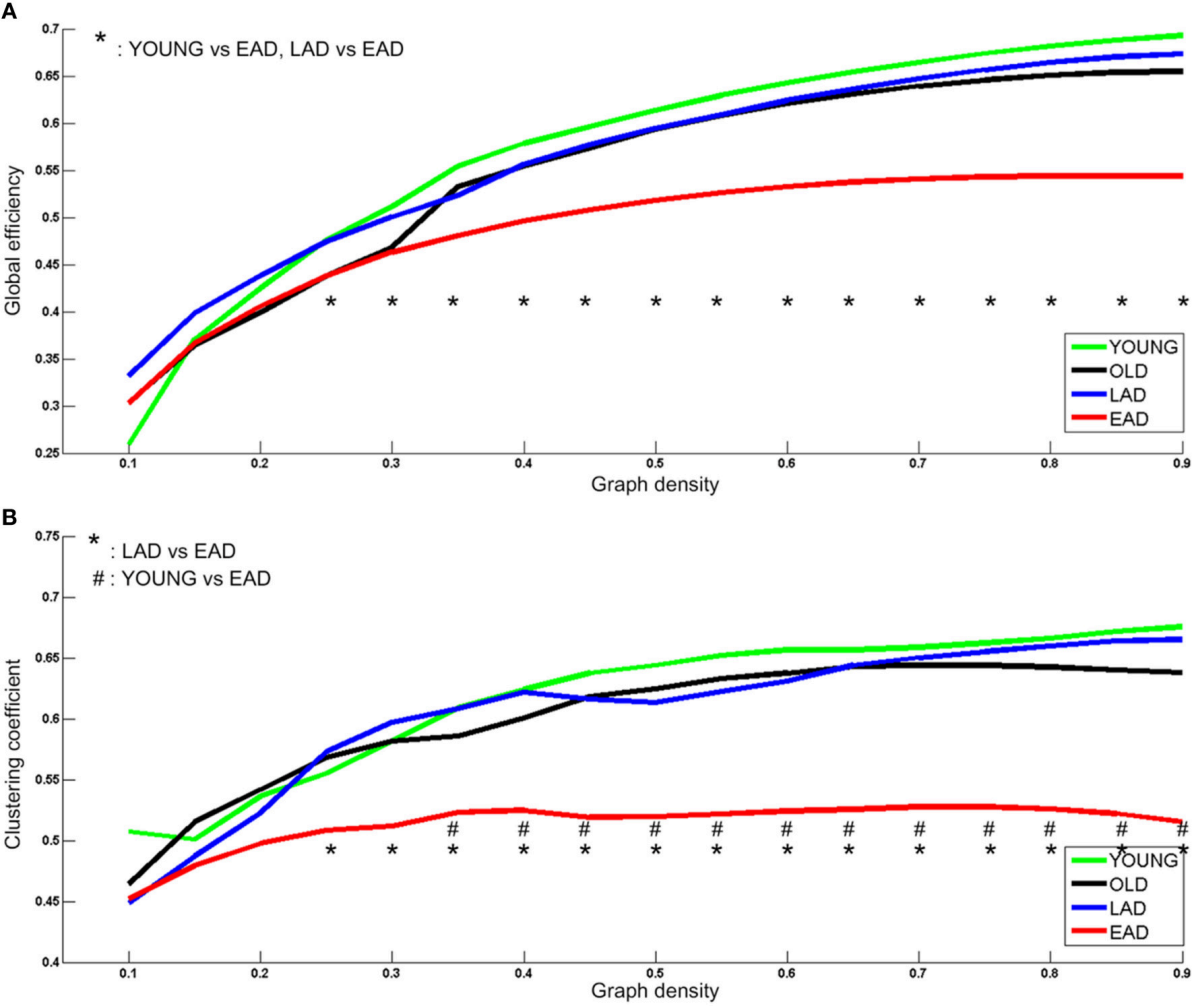
**Metabolic network parameters for Alzheimer's disease (AD) and control groups**. The significant network density period is determined at a threshold of *p* < 0.005 (uncorrected). Each comparison pair is a given different notational value. OLD, old control group; YOUNG, young control group. Global efficiency **(A)** and Clustering coefficient **(B)**.

Network parameters were calculated for each group. Global efficiency and clustering coefficients were not statistically different between the LAD group and old controls (*p* < 0.005, uncorrected) (Figure 5). However, there were significant differences between the young controls and EAD group (*p* < 0.005, uncorrected). The EAD group had a significantly lower global efficiency value than both the young control and LAD groups (25–90%). The clustering coefficient values were also significantly lower in the EAD group compared to the young control (35–90%) and LAD groups (25–90%).

**Figure 6:**
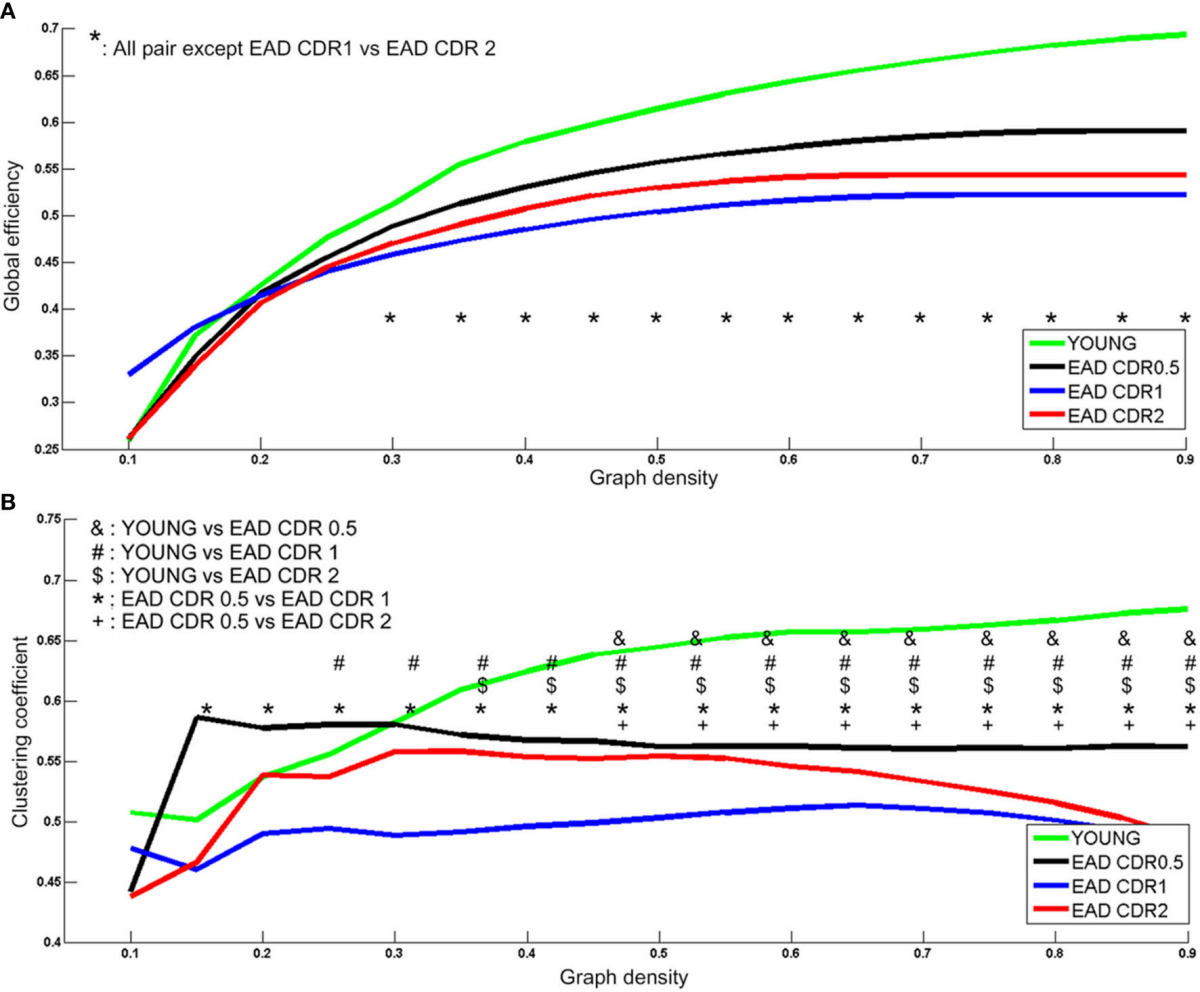
**Metabolic network parameters for the early-onset Alzheimer's disease (EAD) subgroups**. The significant network density period is determined by *p* < 0.005 (uncorrected). Each comparison pair is a given different notational value. YOUNG = young control group. Global efficiency **(A)** and Clustering coefficient **(B)**.

Two network parameters were then compared among the EAD subgroups (Figure 6). Young controls had significantly higher global efficiency values than all the EAD subgroups (30–90%); the CDR = 0.5 subgroup had higher global efficiency values than the other two EAD subgroups (30–90%). There were no differences between the CDR = 1 and CDR = 2 subgroups. Young controls had the highest clustering coefficient compared to each of the EAD subgroups. The CDR = 0.5 subgroup had a higher clustering coefficient than the CDR = 1 (15–90%) and CDR = 2 subgroups (55–90%). The clustering coefficient was not different between the CDR = 1 and CDR = 2 subgroups.

## Discussion

The present study investigated the differences in metabolic connectivity and network parameters between EAD and LAD groups using graph theory analyses. Several studies have examined topographic metabolic changes along with AD progression; however, few investigated metabolic connectivity changes in AD. This study provides additional knowledge regarding the understanding of the pathophysiological differences between LAD and EAD in terms of the metabolic network.

Metabolic connectivity is of interest considering the alterations in brain glucose metabolism in AD. Only few studies have investigated metabolic connectivity associated with this condition. This is mainly because of the limited availability of FDG-PET imaging data. However, the hyperglycemic state associated with diabetes mellitus is a risk factor for AD. This is attributed to the finding that decreased glucose turnover leads to reduced ATP synthesis, eventually bringing about the generation of β-amyloid characteristic to AD (Tuček, 1985; Meier-Ruge et al., 1994). Further, the amyloid protein and insulin are both metabolized by an insulin degradation enzyme in a competitive manner (Haan, 2006). Thus, the characteristics of glucose metabolism could reflect the pathogenesis of AD.

### Metabolic connectivity in EAD and LAD

We first compared the metabolic changes between the EAD and LAD groups against those of age-matched control groups. We hypothesized that actual neural connectivity differences would be reflected in the patterns of metabolic connectivity. In the EAD group, metabolic connectivity was decreased in the anterior cingulate gyrus and occipital area. In the LAD group, connectivity changes were not remarkable, such that differences were only found using less conservative statistical thresholds. These results are consistent with previous studies. For instance, compared to LAD, EAD is characterized by a more severe and broader reduction in resting-state networks (Adriaanse et al., 2014; Gour et al., 2014) and has more distinct symptoms and neuropsychological profiles (Bassett and Bullmore, 2006; Hodges et al., 2006; McMurtray et al., 2006; Shinagawa et al., 2007; Koedam et al., 2009; Migliaccio et al., 2009; Kim et al., 2010; Smits et al., 2012; Van Vliet et al., 2012). Visuospatial dysfunction is regarded as the most distinct neuropsychological feature of EAD. Thus, decreased connectivity between the anterior cingulate gyrus and occipital lobe may be related to the visuospatial dysfunction observed in EAD (Frisoni et al., 2007; Karas et al., 2007; Shiino et al., 2008; Canu et al., 2012). One interesting finding was that the LAD group showed increased connectivity centered on the supplementary motor area at a less conservative statistical threshold. This is in line with a previous study that revealed the supplementary motor area as the most prominent region exhibiting decreased negative connectivity in LAD (Wang et al., 2007). They postulated that the supplementary motor area could be part of a “task-negative network” that degenerates in AD. Thus, the supplementary motor area seems to have increased connectivity because of decreased negative connectivity.

Glucose metabolic connectivity results have also revealed that the degeneration of metabolic connectivity progresses with dementia severity in EAD but not LAD. It is not clear why LAD did not change with the progression of CDR. One possible explanation is that metabolic connectivity is not sufficiently sensitive to detect changes in LAD. Another possibility could be that the changes in metabolic connectivity concurrent with disease progression in LAD are relatively homogenous and widespread; This same explanation could be applicable to the findings in the EAD CDR = 0.5 subgroup, where no statistical differences in metabolic connectivity were observed (*p* < 0.05 FWE corrected), while differences were observed in the EAD CDR = 1 and CDR = 2 subgroups.

Another interesting finding was that CDR = 0.5 subgroup demonstrated increased connectivity in the dorsal cingulate gyrus. The dorsal cingulate gyrus was reported as an atrophic region in EAD (Sakamoto et al., 2002; Ishii et al., 2005; Shiino et al., 2006; Möller et al., 2013). This region is also known as a core region of the DMN that integrates two subnetworks (Buckner et al., 2008). The present study found that metabolic connectivity between the dorsal cingulate gyrus, inferior parietal, and occipital regions decreased significantly. These results are also in line with a previous study revealing that the functional connectivity in the DMN regions was more diminished in EAD compared to LAD (Adriaanse et al., 2014). We assume that decreased connectivity between the dorsal cingulate gyrus and parietal and occipital regions is compensated for by increases in connectivity between the dorsal cingulate gyrus and subcortical regions.

The EAD subgroups had common, diminished connectivity between the occipital and cingulate regions that was further decreased with increased CDR scores. This provides evidence of the degeneration of consecutive metabolic connectivity as dementia progresses. Specifically, increased or decreased connectivity in the dorsal cingulate gyrus was found in the CDR = 0.5 subgroup. These changes were also observed in CDR = 1 and CDR = 2 subgroups at the same threshold. Furthermore, connectivity between the occipital and two (anterior and dorsal) cingulate regions was diminished in the CDR = 1 subgroup. Additionally, connectivity between the occipital and anterior cingulate/medial frontal regions was diminished in the CDR = 2 subgroup. Collectively, this successive degeneration in connectivity indicates that disease progression in EAD could be related with functional decrements in glucose metabolism.

### Network property differences between EAD and LAD

The LAD and EAD groups showed clear differences in terms of metabolic connectivity. First, the two groups differed significantly on small-worldness values. Results indicate that the EAD and young control groups had similar small-worldness values but higher values than the LAD and old control groups. When comparing LAD and old control groups, the LAD group had lower small-worldness values in the 10–25% low-density ranges. Thus, the small-worldness property is preserved in the EAD group differentially from that of the LAD group when compared with age-matched control groups. Secondly, clustering coefficient and global efficiency values for the EAD group were smaller than that of the young control group. The clustering coefficient represents local interconnectivity in a brain network (Watts and Steven, 1998; Rubinov and Sporns, 2010), indicating how much the brain regions are compactly interconnected in a specific module. Thus, a low clustering coefficient value indicates that modularity is low. Conversely, global efficiency represents the efficiency of information transfer in a brain network. A low global efficiency value indicates that the brain network has long functional distances between two regions. Thus, EAD seems to be marked by diminished modularity and network efficiency as a function of disease progression. The present study suggests that EAD shows more severe and localized degeneration within brain networks. Network parameter analyses also revealed successive changes in EAD as a function of disease severity. Thus, decreases in global efficiency and clustering coefficient values track reliably with increases in CDR. However, this was not the case for LAD, where the clustering coefficient and global efficiency values did not show significant differences. The results from the LAD group are consistent with those of previous studies using various neuroimaging modalities, including fMRI and electroencephalogram (EEG). For instance, one EEG study revealed no differences in clustering coefficient values but slight differences in characteristic connectivity paths (Stam et al., 2007). Similarly, a recent fMRI study observed no differences in clustering coefficient values but significant differences in characteristic path lengths at a low density (~20%) (Sanz-Arigita et al., 2010). One possible reason for the lack of differences in LAD network parameters could be that, as previously mentioned, LAD might be associated with relatively homogenous and widespread degeneration.

The connectivity and network results suggest that the dorsal cingulate gyrus, which integrates the DMN subnetworks, is an area where metabolic network degeneration is perhaps initiated, with additional degeneration among other networks as dementia progresses in EAD. However, there was also increased connectivity, presumably indicating compensatory network reorganization, at least during the early stages of EAD.

### Conclusions

To the best of our knowledge, this is the first study comparing glucose metabolic connectivity and network properties in EAD and LAD. One particular novel finding of this study is the delineation of distinct metabolic networks in EAD and its subgroups. The main findings can be summarized as follows: (i) EAD and LAD have differences in glucose metabolic connectivity; (ii) EAD subgroups show progressive degeneration in glucose metabolic connectivity, mainly between the cingulate gyri and occipital regions; (iii) clustering coefficient and global efficiency values decrease significantly in EAD compared to age-matched control groups, but did not differ from those of the LAD group; and (iv) network parameters gradually decreased as a function of dementia progression. Thus, it appears that EAD and LAD have distinct features in terms of metabolic connectivity and metabolic network properties. These new findings may suggest that we may need different approach in detection and probably in treatment of the EAD patients.

## Author contributions

JC: the design of the work, analysis and interpretation of data for the work, drafting the work, final approval of the version to be published, and agreement to be accountable for all aspects of the work. KY: interpretation of data for the work and revising the work critically for important intellectual content, final approval of the version to be published, and agreement to be accountable for all aspects of the work. EK: interpretation of data for the work, the acquisition of data, revising the work critically for important intellectual content, final approval of the version to be published, and agreement to be accountable for all aspects of the work. DN: interpretation of data for the work, the acquisition of data, revising the work critically for important intellectual content, final approval of the version to be published, and agreement to be accountable for all aspects of the work. YJ: the conception of the work, drafting the work, revising the work critically for important intellectual content, final approval of the version to be published and agreement to be accountable for all aspects of the work.

### Conflict of interest statement

The authors declare that the research was conducted in the absence of any commercial or financial relationships that could be construed as a potential conflict of interest.
